# Outcome of Root Canal Treatment of Necrotic Teeth with Apical Periodontitis Filled with a Bioceramic-Based Sealer

**DOI:** 10.1155/2021/8816628

**Published:** 2021-03-18

**Authors:** Kawther Bel Haj Salah, Sabra Jaâfoura, Mahdi tlili, Marwa Ben Ameur, Saida Sahtout

**Affiliations:** ^1^Department of Conservative Odontology, Laboratory of Dento-Facial Clinical and Biological Approach (ABCDF) LR12ES10, Faculty of Dental Medicine, University of Monastir, Avicenne Avenue, Monastir 5019, Tunisia; ^2^Department of Dental Biomaterials, Laboratory of Dento-Facial Clinical and Biological Approach (ABCDF) LR12ES10, Faculty of Dental Medicine, University of Monastir, Avicenne Avenue, Monastir 5019, Tunisia; ^3^DMD, Private Practice in Sahloul-Sousse, Laboratory of Dento-Facial Clinical and Biological Approach (ABCDF) LR12ES10, Faculty of Dental Medicine, University of Monastir, Avicenne Avenue, Monastir 5019, Tunisia

## Abstract

**Introduction:**

Apical periodontitis is among the most common pathologies in endodontics. The treatment of apical periodontitis has always been an important occupation in the modern practice of endodontics, and the failure has been associated with nonhermetic root canal filling. With that in mind, bioceramic-based sealers have been incorporated into endodontic practice. The purpose of this study was to evaluate the outcome of nonsurgical root canal treatment (RCT), using a single-cone and Bioroot RCS filling of necrotic teeth with apical periodontitis.

**Materials and Methods:**

This follow-up study included patients treated in the department of Restorative Dentistry and Endodontics in the Dental Clinic of Monastir, from January 2018 to December 2019. The study intended to include all adult patients presenting a symptomatic or asymptomatic apical periodontitis. Once the diagnosis was performed, the patients were divided into two groups: a one-session treatment group and a two-session treatment group. All cases were obtured with BioRoot using a single-cone technique with a minimum of a 6-month recall. At 6-month follow-ups, teeth were classified as healed, healing (success), or not healed (failure), based on clinical and radiographic findings.

**Results:**

Twelve patients met the inclusion criteria, six patients per group. Seven patients returned for follow-ups. At 6-month follow-ups, the overall success rate was 100%, with 57.1% determined to be “healed” and 42.8% determined to be “healing.” All the PAI scores decreased compared to the baseline situation.

**Conclusion:**

The results obtained showed the contribution of BioRoot RCS in the healing of periapical lesions. Accordingly, bioceramic-based sealers seem to optimize the prognosis of root canal treatments.

## 1. Introduction

Apical periodontitis is among the most common pathologies in endodontics. It is usually associated with periradicular bone alterations, which could be identified in periapical X-rays [1]. The treatment of apical periodontitis has always been an important occupation in the modern practice of endodontics. Research has described a success rate of up to 87% [2], and the failure has been associated with nonhermetic root canal filling [3]. Sealing ability and bioactivity are interesting proprieties to enhance the success of root canal treatment [4].

Bioceramic-based sealers have been incorporated into endodontic practice to improve the outcome of root canal treatment [5]. BioRoot™ RCS (Septodont) is a calcium-silicate-based sealer, which has been marketed since 2015. It is composed of zirconium oxide, tricalcium silicates, and water-soluble polymer [6]. It demonstrated several desirable properties such as biocompatibility, chemical stability, flowability, hydrophilicity, biomineralization, calcium ion release, and hydroxyapatite formation. Thus, these qualities improve the hermiticity of root canal obturation and, hence, the healing of periapical lesions.

One of the significant strides in root canal treatment is to make a well-sealed root canal framework. The recent advent of a bioceramic-based endodontic sealer promoted the single-cone obturation technique. In fact, Chybowski et al. found that a hydraulic cement (EndoSequence BC Sealer) used with a single-cone technique is a viable option for obturation [7]. Besides, BioRoot™ RCS in combination with single cone resulted in a comparable success rate compared to a warm vertical condensation with AH plus [8].

Up to now, no studies have been carried out in Tunisia reporting the effectiveness of Bioroot RCS. Accordingly, this follow-up study is considered as a pilot study in our country. The following study aims to evaluate the feasibility to run a large trial on the outcome of nonsurgical root canal treatment (NSRCT) using a bioceramic sealer in a single-cone technique in cases of apical periodontitis.

## 2. Case Presentation

### 2.1. Case Selection and Treatment Procedure

This follow-up study is a monocentric analytical case series. Study subjects were recruited from the pool of patients attending the department of Restorative Dentistry and Endodontics in the Dental Clinic of Monastir, from January 2018 to December 2019. The study intended to include all adult patients presenting a symptomatic or asymptomatic apical periodontitis. All patients were selected according to the criteria developed in Table 1.

The diagnosis was obtained by clinical and radiographic manifestations as well as the results of the different tests executed in a routine dental investigation. At that point, the practitioner decides whether to perform the root canal treatment in a single session or in two sessions. Accordingly, patients were divided into two groups: a one-session treatment group and a two-session treatment group. The size of the lesion would be assessed in the intended trial at baseline and at follow-up.

All treatments were performed by a third-year endodontic resident following a standardized treatment protocol. Once written and verbal informed consent was obtained, local anesthesia was administered, if needed, for patient comfort and a rubber dam was applied. The access cavity was prepared, and the canals were shaped using the Protaper Next (Dentsply, Maillefer) up to the finishing file x2 (25/06). The working length was determined using an electronic apex locator (Locapex FIVE; Ionix, FR) and confirmed by using a straight and angled radiograph. Throughout instrumentation, a 3.25% NaOCl irrigation solution was administered copiously followed by a final rinse with 3 ml 17% EDTA (METABIOMED) for 2 minutes. Passive manual activation was used to aid irrigation, and then, canals were dried with paper points. Teeth in group 1 were filled in the same session: BioRoot RCS was mixed following the manufacturer's recommendations, the canal walls were coated with BioRoot RDC using a paper point, and a gutta-percha point was tipped into the mixed material and gently inserted until reaching the working length. For teeth assigned to group 2, calcuim hydroxide was placed into the canals and the access cavities were sealed with zinc oxide-eugenol cement. At the second appointment, the canals were once again debrided and dried and then filled with the same protocol described for group 1. After completion of treatment, the teeth were restored with composite resin (Z350 3M ESPE). The patients were recalled 3 months and 6 months after treatment until complete healing of the periapical lesion.

Ethical approval was obtained (Ethics Clearance Certificate on 10/01/2018) by the Chairwoman and Professor Nadia Frih from the Human Research Ethics Committee (Medical), University of Monastir.

### 2.2. Calibration

Two investigators evaluated all the radiographic images independently; each investigator already graded a set of 20 periapical radiographs not linked to the study. These periapical radiographs were chosen for training ends. It worked as a normalization exercise for using the Digimizer 4.3 and PAI scoring system [9]. In 83% of cases, agreement was obtained. In cases with discordance, a third examiner was consulted, and joint reevaluation was performed until a compromise was reached.

### 2.3. Clinical and Radiographic Evaluation

Patients were scheduled for recall examination to assess the clinical and radiographic healing response. The examination was documented and included any clinical signs or symptoms. Periapical radiographs were taken using the same parameters and then evaluated by the calibrated examiners.

The measurements of the lesion (area in mm^2^) were performed using Digimizer 4.3. Then, the severity was determined by the periapical index (PAI) scoring system described by Orstavik et al. [10].

### 2.4. Outcome Assessment

Treated teeth were divided into outcome categories according to the following classification:

1. Healed: fully functional and asymptomatic teeth with no or minimal periradicular radiolucency, accompanied by PAI scores of 1 or 2
2. Healing: functional teeth and clinical normalcy other than sensitivity to percussion, with decrease in periradicular radiolucency and in the PAI scores
3. Nonhealed: nonfunctional symptomatic teeth with an increased or unchanged size of the radiolucency

The outcome of endodontic treatment was classified as a success if lesions are healed or healing and as a failure if lesions are unhealed. All variable parameters have been examined. Patient-related factors are sex, health, and age. Tooth factors are lesion size, tooth type, and preoperative symptoms. Treatment factors included treatment protocol (one visit or two visits), sealer extrusion, and follow-up time.

### 2.5. Analysis of Data

After the quantification of periapical lesions (PAI scores and Digimizer), the data were grouped and analyzed using Microsoft Excel.

### 2.6. Results

**Figure 1:**
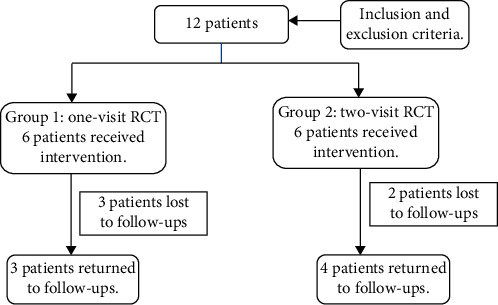
Diagram showing patients' selection.

Twelve patients met the inclusion criteria, six patients per group. Seven patients returned for follow-ups: three patients in the one-visit group (group 1) and four patients in the two-visit group (group 2). Three patients reached an 18-month follow-up, and four patients attained a 6-month follow-up and are still under control until complete healing of the periapical lesion (Figure 1). For the consistency of results, PAI scores were compared at 6-month follow-ups. The demographic information and the distribution of patients within the two groups are given in Table 2.

**Figure 2:**
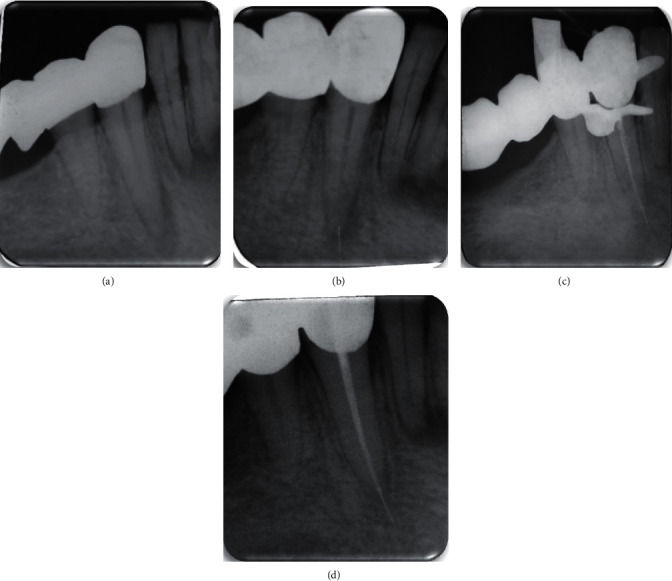
A case of a healed lesion: (a) preoperative radiograph; (b) radiograph showing the fracture of the K20 file; (c) postoperative radiograph; and (d) a 6-month follow-up radiograph.

**Figure 3:**
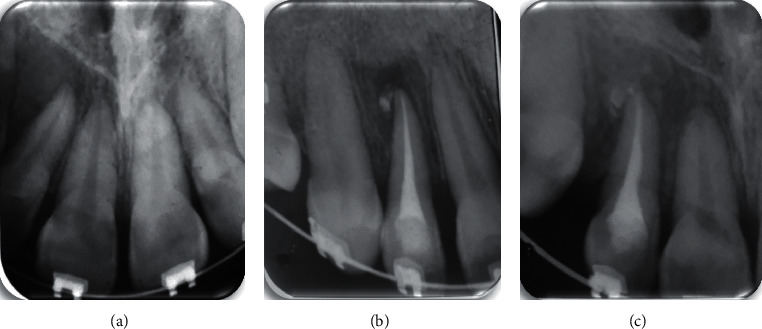
A case of a healing lesion: (a) preoperative radiograph; (b) postoperative radiograph showing the extrusion of Bioroot RCS; and (c) a 6-month follow-up radiograph.

At 6-month follow-ups, the overall success rate was 100%, with 57.1% determined to be “healed” with a complete healing of the periapical lesion and 42.8% determined to be “healing” (Figures 2 and 3 ). All the preoperative PAI scores were 4 except one which was 5. During the recall appointments, the PAI scores decreased compared to the baseline situation (Table 3).

In group 1, one out of three lesions could be considered completely healed after 6-month follow-up, and in group 2, three out of four lesions could be considered healed (Table 2).

### 2.7. Sample Size Calculation for a Future Randomized Trial

The sample size was determined based on not only the confidence level with which we would like to detect the outcome of nonsurgical root canal treatment using BioRoot Rcs in a single-cone technique but also the actual probability that withdrawal/dropouts manifest themselves in a potential study participant. At a 95% confidence level with *π* = .05 probability, the trial will require the inclusion of 150 teeth [11].

## 3. Discussion

The use of BioRoot RCS both in one- and two-session treatment showed satisfying outcomes. The characteristics of root canal sealers have a huge impact on the quality of the root canal filling [9]. BioRoot RCS creates an alkaline environment by raising the pH [6, 12] which could be caused by the Ca^2+^ and OH^−^ leaching. The alkalinizing activity and the Ca^2+^ release prevents bacterial proliferation and stimulates periodontal and endodontic tissue regeneration. Besides, it enhances the biocompatibility and the bioactivity of the sealer [13].

Many endodontic sealers showed some toxicity in their freshly mixed state [14]. BioRoot RCS showed a nonsignificant cytotoxicity. Compared to current root canal sealers (AHPlus, Endorez, Acroseal, and MTA Fillapex), it has the lowest genotoxicity and cytotoxicity in periodontal ligament stem cells [15] and showed an excellent biocompatibility [16]. BioRoot is not only biocompatible but also bioactive through its capacity to induce the secretion of hard tissue both in the bone and in the dental pulp [17]. This bioactivity is a result of the interaction of calcium hydroxide produced as a reaction product of tricalcium silicate hydration with phosphates present in tissue fluids [18, 19]. So, along the root canal walls, BR interacts with the dentin. When in contact with the physiologic fluid, it releases calcium and forms an interfacial calcium phosphate layer, and consequently, it develops a chemical bond with dentinal walls [18].

BioRoot has a great biomineralization effect, which results from the interaction of the BR with dentinal fluid. Mineral plugs within dentinal tubules are observed [20], thus establishing a barrier to prevent passage of microorganisms from the root canal system to the periapical tissues and vice versa [14].

It is a well-known fact that warm compaction and cold lateral compaction of the gutta-percha are among the most commonly used obturation techniques [21]. When using BioRoot RCS, manufacturers recommend the cold lateral condensation technique [18] and, more precisely, the single-cone technique. Indeed, during the warm vertical compaction technique for BioRoot RCS, the application of heat affects the sealer physical properties [22]; the setting time is affected due to evaporation of warm water from the water-based sealer, but no chemical changes happened [18]. Angelo et al. proved, in a nonrandomized clinical trial conducted in 2020 on 150 teeth, at one-year recall, obturation with a bioceramic-based sealer. Also, the single-cone technique presented a similar success rate compared to the warm vertical condensation technique at 12 months [8].

The gutta-percha cones cannot seal the root canal, and they act only as a filler [23]. Actually, with the mineral-based root canal sealers, the paradigm has changed; the sealer has become the main body of the filling, and the gutta-percha cone serves only as a post.

It is worth noting that the root canal preparation is one of the most important steps in a successful root canal treatment [24]. Root canal shaping was performed with Protaper Next (Denstply, Sirona) (PTN). This provided a shorter instrumentation time than with Protaper Universal (PU) [25]. According to Karatas et al. [24], PTN created significantly fewer dentinal cracks than PU and Wave-One systems in the apical third. In addition to that, Topçuoglu et al. [26] suggested that preparing the canal with Protaper Next files was associated with less extruded apical debris when compared to Protaper Universal and Reciproc files (VDW). This apically extruded debris, formed during root canal instrumentation, might induce flare up and, perhaps, root canal treatment failure [27]. Therefore, it must be reduced to make a better outcome of endodontic treatment.

Since the main critical goal of root canal treatment is the eradication or the significant reduction and control of the bacterial population to ensure periradicular tissue healing of the apical periodontitis [28], it can be guaranteed only if mechanical instrumentation is supported by chemical irrigation [29]. In fact, the irrigant will flush out debris, lubricate the dentinal walls, dissolve organic components, and reduce bacterial load in the root canal [30]. In several studies, authors reported that 1–5% sodium hypochlorite (NaOCl) solutions have an important spectrum antimicrobial activity [31]. On top of that, Baumgartner and Cuenim [32] suggested that 1%, 2.5%, and 5.25% sodium hypochlorite could remove pulpal remnants and predentin from uninstrumented areas. Also, it is well known that an increase in NaOCl solution concentration offers an increase in the antibacterial activity [33]. This antimicrobial property is always improved by copiously irrigating using larger volumes [34].

The outcome of root canal therapy is based on clinical and radiological data. Since the clinical symptoms were absent at the check-up appointment, the outcome was judged based on the radiographic assessment.

Small samples may be appropriate for a pilot demonstrating the ability to execute a specific research protocol. Piloting a study on a smaller scale can help to identify unforeseen problems that could compromise the quality or flow of the study [11].

At 6-month follow-ups, all the clinical cases presented neither the expansion of the periapical radiolucency area nor the appearance of new rarefactions; contrarily, an increase in bone density and a decrease of intrabecular spaces were observed. The treatment outcome was considered as “success” in 100% of cases: 4 cases were determined to be healed with a complete resolution of the periapical lesion, and 3 cases were determined to be healing.

The success rate for healing in this study is higher than the medium success rate found in other studies that assess the success rate of nonsurgical endodontic therapy of teeth with periapical lesions. Moazami et al. [35] have had a success rate of about 87% in teeth with periapical lesions, while Peters et al. have had a success rate of about 75% in 115 teeth with periapical lesion; 20% lower than the cases without lesions [36].

In this paper, the recall radiographs showed bone thickening and relative or complete resolution of the periapical radiolucency: the mineral loss was progressively occupied with bone, and the radiographic density increased within the lesion. These findings are consistent with the properties of BioRoot™ RCS. In fact, it has a great adhesion to the gutta-percha and dentin and a great flowability, so it provides hermetic obturation of the apical foramen, isthmus, and accessory canals [37]. Camps et al. [17] suggested that BioRoot has the potential to induce angiogenesis and osteogenesis. These properties are essential for periapical tissue regeneration.

For this study, the cases were divided into two groups, cases treated in one session and cases treated in two sessions. Both groups showed a decrease in PAI scores at 6-month follow-ups. There was no significant difference between groups. These results are consistent with the results of Penesis et al. [38] and the work of Peters and Wesselink [39] showing no correlation between healing of endodontic lesions and treatment in one or two visits with interappointment dressing of calcium hydroxide.

Even though many authors believe that treatment with an intracanal dressing of calcium hydroxide would improve bone healing [40], other investigators instead believe that calcium hydroxide is not effective on all endodontic pathogens and, therefore, does not provide a real contribution when used in multiple-appointment root canal therapy [41–44]. Our findings are in harmony with the majority of clinical studies showing no statistically significant differences between one-visit and two-visit treatments with an interappointment calcium hydroxide paste [39, 45, 46].

On the other hand, many authors [39, 47] have suggested that 4 or 5 years are necessary to evaluate healing on teeth with periapical lesions. Penesis et al. [38] demonstrated that 12 months might be ideal to control periapical changes in bone density when using PAI scores. Finally, according to Bystrom et al. [48], as long as there is a reduction in the size of the lesion's size when filled with BioRoot™RCS, no more follow-ups are needed. In the present study, the decrease in the size of the lesion, the decrease of PAI scores, the change in radiodensity within the lesions, and the appearance of the trabecular bone could indicate successful treatment and the healing of the periapical lesion. It is important to mention that follow-up periods are still ongoing for some of the clinical cases till complete healing.

## 4. Conclusions

The current study monitors the progress of the healing process of periapical lesions after root canal treatment, using a bioceramic-based sealer, the Bioroot™ RCS. The results obtained showed the contribution of BioRoot RCS in the healing of periapical lesions. Accordingly, bioceramic-based sealers, although lacking clinical experience, seem to optimize the prognosis of root canal treatments. A clinical trial in a large scale must be considered to assess the potential systematic indication of the bioceramic-based sealer in a single-cone technique in cases of apical periodontitis.

## Figures and Tables

**Table 1:** Inclusion and exclusion criteria.

Inclusion criteria	Exclusion criteria
A mature tooth	Poor oral hygiene
Cooperative patient	Reserved tooth prognosis
Symptomatic or asymptomatic apical	Patient with multimorbidity
Periodontitis	Patient refusing to come back to the recall
Periradicular radiolucency	Unwilling patients or those who could not be contacted

**Table 2:** Patient characteristics and outcome assessment.

Groups	Patient	Age	Preoperative symptoms	Sealer extrusion	Follow-up time (months)	PAI_0_	PAI_f_^∗^	Rating∗	Outcome
Group 1	1	17	No	Yes	18	4	2	Healing	Success
	2	59	No	No	6	4	1	Healed	Success
	3	28	Yes	No	6	5	3	Healing	Success
Group 2	4	54	Yes	No	18	4	1	Healed	Success
	5	28	Yes	No	6	4	3	Healing	Success
	6	25	No	No	6	4	1	Healed	Success
	7	57	No	Yes	18	4	2	Healed	Success

PAI_f_^∗^: PAI score at 6-month follow-up. Rating∗: at a 6-month follow-up.

**Table 3:** Description of the PAI scoring system [10].

PAI	Description
1	Normal periapical structure
2	Small changes in the bone structure
3	Changes in the bone structure with some mineral loss
4	Periodontitis with a well-defined radiolucent area
5	Severe periodontitis with exacerbating features

## Data Availability

The data used to support the findings of this study are included within the article.

## References

[1] Sigurdsson A., Garland R. W., Le K. T., Rassoulian S. A. (2018). Healing of periapical lesions after endodontic treatment with the GentleWave procedure: a prospective multicenter clinical study. *Journal of Endodontics*.

[2] Fidgor D. (2002). Apical periodontitis: a very prevalent problem. *Oral Surgery, Oral Pathology, Oral Radiology, and Endodontology*.

[3] Gadzhula N. H. (2016). Clinical effictiveness of treatment the patients with chronic apical periodontitis. *International Journal of Medical Research*.

[4] Kikly A., Jaâfoura S., Kammoun D., Sahtout S. (2020). Sealing ability of endodontic cements: an in vitro study. *International Journal of Dentistry*.

[5] Jain P., Ranjan M. (2015). The rise of bioceramics in endodontics: a review. *International Journal of Pharma and Bio Sciences*.

[6] Khalil I., Naaman A., Camilleri J. (2016). Properties of tricalcium silicate sealers. *Journal of Endodontics*.

[7] Chybowski E. A., Glickman G. N., Patel Y., Fleury A., Solomon E., He J. (2018). Clinical outcome of non-surgical root canal treatment using a single-cone technique with endosequence bioceramic sealer: a retrospective analysis. *Journal of Endodontics*.

[8] Angelo Z., Knight A., Federico F., Francesco M. (2020). Outcome of RootCanal treatments using a new calcium silicate root canal sealer: a non-randomized clinical trial. *Journal of Clinical Medicine*.

[9] Rai R., Singbal K., Parekh V. (2016). The effect of temperature on rheological properties of endodontic sealers. *Journal of Conservative Dentistry*.

[10] Orstavik D., Kerekes K., Eriksen H. M. (1986). The periapical index: a scoring system for radiographic assessment of apical periodontitis. *Dental Traumatology*.

[11] Viechtbauer W., Smits L., Kotz D. (2015). A simple formula for the calculation of sample size in pilot studies. *Journal of Clinical Epidemiology*.

[12] Siboni F., Taddei P., Zamparini F., Prati C., Gandolfi M. G. (2017). Propreties of BioRoot RCS, a tricalcium silicate endodontic sealer modified with povidone and polycarboxylate. *International Endodontic Journal*.

[13] Matsumoto S., Hayashi M., Suzuki Y., Suzuki N., Maeno M., Ogiso B. (2013). Calcium ions released from mineral trioxide aggregate convert the differentiation pathway of C2C12 cells into osteoblast lineage. *Journal of Endodontics*.

[14] Jung S., Sielker S., Hanisch M. R., Libricht V., Schafer E., Dammaschke T. (2018). Cytotoxic effects of four different root canal sealers on human osteoblasts. *PLoS One*.

[15] Eldeniz A. U., Shehata M., Högg C., Reichl F. X. (2016). DNA double-strand breaks caused by new and contemporary endodontic sealers. *International Endodontic Journal*.

[16] Raghavendra S. S., Jadhav G. R., Gathani K. M., Kotadia P. (2017). Bioceramics in endodontics-a review. *Journal of Istanbul University Faculty of Dentistry*.

[17] Camps J., Jeanneau C., El Ayachi I., Laurent P., About I. (2015). Bioactivity of a calcium silicate-based endodontic cement (BioRoot RCS): interactions with human periodontal ligament cells in vitro. *Journal of Endodontics*.

[18] Camilleri J. (2015). Sealers and warm gutta-percha obturation techniques. *Journal of Endodontics*.

[19] Tay F. R., Pashley D. H., Rueggeberg F. A., Loushine R. J., Weller R. N. (2007). Calcium phosphate phase transformation produced by the interaction of the portland cement component of white mineral trioxide aggregate with a phosphate-containing fluid. *Journal of Endodontics*.

[20] Reyes-Carmona J. F., Felippe M. S., Felippe W. T. (2010). A Phosphate-buffered saline intracanal dressing improves the biomineralization ability of mineral trioxide aggregate apical plugs. *Journal of Endodontics*.

[21] Moinzadeh A. T., Zerbst W., Boutsioukis C., Shemesh H., Zaslansky P. (2015). Porosity distribution in root canals filled with gutta percha and calcium silicate cement. *Dental Materials*.

[22] Qu W., Bai W., Liang Y.-H., Gao X.-J. (2016). Influence of warm vertical compaction technique on physical properties of root canal sealers. *Journal of Endodontics*.

[23] Trope M., Bunes A., Debelian G. (2015). Root filling materials and techniques: bioceramics a new hope?. *Endodontic Topics*.

[24] Karatas E., Gunduz H. A., Kirici D. O., Arslan H., Topçu M. C., Yeter K. Y. (2015). Dentinal crack formation during root canal preparations by the twisted file adaptive, protaper next, protaper universal, and waveone instruments. *Journal of Endodontics*.

[25] Capar I. D., Arslan H., Akcay M., Ertas H. (2014). An in vitro comparison of apically extruded debris and instrumentation times with protaper universal, protaper next, twisted file adaptive, and hyflex instruments. *Journal of Endodontics*.

[26] Topçuoglu H. S., Zan R., Akpek F. (2016). Apically extruded debris during root canal preparation using Vortex Blue, K3XF, Protaper Next and Reciproc instruments. *International Endodontic Journal*.

[27] Zhang C., Liu J., Liu L. (2018). The influence of protaper and WaveOne on apically extruded debris: a systematic review and meta-analysis. *Journal of conservative dentistry*.

[28] Siqueira J. F., Rôças I. N. (2008). Clinical implications and microbiology of bacterial persistence after treatment procedures. *Journal of Endodontics*.

[29] Sjögren U., Hägglund B., Sundqvist G., Wing K. (1990). Factors affecting the long-term results of endodontic treatment. *Journal of Endodontics*.

[30] Navarro-Escobar E., González-Rodríguez M. P., Ferrer-Luque C. M. (2010). Cytotoxic effects of two acid solutions and 2.5% sodium hypochlorite used in endodontic therapy. *Medicina Oral, Patología Oral y Cirugía Bucal*.

[31] Siqueira J. F., Rôças I. N., Favieri A., Lima K. C. (2000). Chemomechanical reduction of the bacterial population in the root canal after instrumentation and irrigation with 1%, 2.5%, and 5.25% sodium hypochlorite. *Journal of Endodontics*.

[32] Baumgartner J. C., Cuenin P. R. (1992). Efficacy of several concentrations of sodium hypochlorite for root canal irrigation. *Journal of Endodontics*.

[33] Berber V. B., Gomes B. P. F. A., Sena N. T. (2006). Efficacy of various concentrations of NaOCl and instrumentation techniques in reducing *Enterococcus faecalis* within root canals and dentinal tubules. *International Endodontic Journal*.

[34] Bronnec F., Bouillaguet S., Machtou P. (2010). Ex vivo assessment of irrigant penetration and renewal during the final irrigation regimen. *International Endodontic Journal*.

[35] Moazami F., Sahebi S., Sobhnamayan F., Alipour A. (2011). Success rate of nonsurgical endodontic treatment of nonvital teeth with variable periradicular lesions. *Iranian Endodontic Journal*.

[36] Peters O. A., Barbakow F., Peters C. I. (2004). An analysis of endodontic treatment with three nickel-titanium rotary root canal preparation techniques. *International Endodontic Journal*.

[37] Simon S., Flouriot A. C. (2016). BioRoot™ RCS a new biomaterial for root canal filling. *Journal of Case Studies Collection*.

[38] Penesis V. A., Fitzgerald P. I., Fayad M. I., Wenckus C. S., BeGole E. A., Johnson B. R. (2008). Outcome of one-visit and two-visit endodontic treatment of necrotic teeth with apical periodontitis: a randomized controlled trial with one-year evaluation. *Journal of Endodontics*.

[39] Peters L. B., Wesselink P. R. (2002). Periapical healing of endodontically treated teeth in one and two visits obturated in the presence or absence of detectable microorganisms. *International Endodontic Journal*.

[40] Fernandes M., Ataide I. (2010). Nonsurgical management of periapical lesions. *Journal of Conservative Dentistry*.

[41] Siqueira J. F., Lopes H. P. (1999). Mechanisms of antimicrobial activity of calcium hydroxide: a critical review. *International Endodontic Journal*.

[42] Dahlén G., Samuelsson W., Molander A., Reit C. (2000). Identification and antimicrobial susceptibility of enterococci isolated from the root canal. *Oral Microbiology and Immunology*.

[43] Sathorn C., Parashos P., Messer H. (2007). Antibacterial efficacy of calcium hydroxide intracanal dressing: a systematic review and meta-analysis. *International Endodontic Journal*.

[44] Sundqvist G., Figdor D., Persson S., Sjögren U. (1998). Microbiologic analysis of teeth with failed endodontic treatment and the outcome of conservative re-treatment. *Oral Surgery, Oral Medicine, Oral Pathology, Oral Radiology, and Endodontology*.

[45] Trope M., Delano E. O., Ørstavik D. (1999). Endodontic treatment of teeth with apical periodontitis: single vs. multivisit treatment. *Journal of Endodontics*.

[46] Molander A., Warfvinge J., Reit C., Kvist T. (2007). Clinical and radiographic evaluation of one- and two-visit endodontic treatment of asymptomatic necrotic teeth with apical periodontitis: a randomized clinical trial. *Journal of Endodontics*.

[47] Weiger R., Rosendahl R., Lost C. (2000). Influence of calcium hydroxide intracanal dressings on the prognosis of teeth with endodontically induced periapical lesions. *International Endodontic Journal*.

[48] Bystrom A., Happonen R.-P., Sjogren U., Sundqvist G. (1987). Healing of periapical lesions of pulpless teeth after endodontic treatment with controlled asepsis. *Dental Traumatology*.

